# Feasibility, safety, and acceptability of intranasal heroin-assisted treatment in Switzerland: protocol for a prospective multicentre observational cohort study

**DOI:** 10.1186/s13722-023-00367-0

**Published:** 2023-03-11

**Authors:** Jean N. Westenberg, Maximilian Meyer, Johannes Strasser, Michael Krausz, Kenneth M. Dürsteler, Luis Falcato, Marc Vogel

**Affiliations:** 1 University of Basel Psychiatric Clinics, Wilhelm Klein-Strasse 27, 4002 Basel, Switzerland; 2 Department of Psychiatry, Faculty of Medicine, University of British Columbia, Vancouver, BC Canada; 3 School of Medicine, University College Cork, Cork, Ireland; 4 Department of Psychiatry, Psychotherapy and Psychosomatics, Psychiatric Hospital, University of Zürich, Zurich, Switzerland; 5 Arud Zentrum Für Suchtmedizin, Zurich, Switzerland

**Keywords:** Heroin, Diacetylmorphine, Protocol, Prospective study, Observational study, Opioid use disorder

## Abstract

**Background:**

Heroin-assisted treatment (HAT) is a proven effective treatment option for individuals with severe opioid use disorder (OUD). In Switzerland, pharmaceutical heroin (diacetylmorphine, DAM) is available in tablet form or as injectable liquid. This creates a large barrier for individuals who require the rapid onset of effect but are either unable or do not want to inject, or who primarily snort opioids. Early experimental data has demonstrated that intranasal DAM administration can be a viable alternative to the intravenous or intramuscular route of administration. The purpose of this study is to assess the feasibility, safety, and acceptability of intranasal HAT.

**Methods:**

This study will assess intranasal DAM using a prospective multicentre observational cohort study design in HAT clinics across Switzerland. Patients will be offered to switch from oral or injectable DAM to intranasal DAM. Participants will be followed-up over 3 years, with assessments at baseline, and after 4, 52, 104 and 156 weeks. The primary outcome measure (POM) is retention in treatment. Secondary outcomes (SOM) include prescriptions and routes of administration of other opioid agonists, illicit substance use, risk behaviour, delinquency, health and social functioning, treatment adherence, opioid craving, satisfaction, subjective effects, quality of life, physical health, and mental health.

**Conclusions:**

The results derived from this study will generate the first major body of clinical evidence on the safety, acceptability, and feasibility of intranasal HAT. If proven to be safe, feasible and acceptable, this study would increase the accessibility of intranasal OAT for individuals with OUD globally as a critical improvement in risk reduction.

## Introduction

Heroin-assisted treatment (HAT) is an established and effective treatment option for individuals with opioid use disorder (OUD) that do not respond to conventional opioid agonist treatment (OAT), namely methadone, buprenorphine and slow-release oral morphine [1, 2]. HAT includes the supervised provision of oral or injectable (intravenous; IV) pharmaceutical heroin (diacetylmorphine, DAM). HAT recognises that some patients require a rapid onset of opioid effects, in particular euphoria, which is associated with the subjective experience of a “rush” or “high” [3].

However, currently available routes of DAM administration (IV, oral) are increasingly insufficient in an aging user population. In Switzerland, individuals with OUD, including HAT patients, are ageing [4, 5]. With age and a long history of controlled IV DAM use or uncontrolled IV illicit opioid use, the state of access-veins deteriorates. As such, patients that are no longer able to inject DAM into peripheral veins due to deterioration either revert to injection in the inguinal veins/groin [6], to intramuscular injection, subcutaneous injection or change to oral DAM tablets. All these alternatives may be associated with reduced treatment outcomes. For instance, groin injections have been associated with complications such as deep vein thrombosis, mis-injection into artery or nerve, aneurism or severe infections and abscesses [6]. Similarly, subcutaneous (SC) and intramuscular (IM) injections have been related to possibly life-threatening injection-related injuries and diseases [7, 8]. Clinical experience shows that IM or SC injection is associated with complications such as infections and abscesses, indurations or skin lesions and is often described as painful [4, 9]. Furthermore, onset of effect and subjective “flash” are usually described as slower and less strong when injecting IM or SC. Both may also be contraindicated for persons on oral anticoagulation because of the risk of bleeding and haematoma. Alternatively, switching from IV DAM to oral DAM treatment is associated with fewer complications. However, oral DAM has a much slower absorption, lower bioavailability and less-strong onset of effect than IV but also IM and likely SC injecting [10–12]. It therefore produces only a relatively mild “rush” or euphoria, which renders oral DAM undesirable for many HAT patients.

Currently available routes of DAM administration (IV and oral) are also increasingly insufficient due to changing patterns of substance use. In Switzerland and the EU, intranasal use is increasing, while inhalation and injection are decreasing [13, 14]. Given the current treatment options, individuals who are using opioids intranasally and entering HAT are prescribed oral DAM. A prescription of injectable DAM for non-injecting patients would ethically be unacceptable due to the higher risks associated with this route of administration. However, as previously mentioned, oral DAM has a much slower onset and less subjective effects. Hence, it often has limited efficiency in this patient group and may lead to treatment drop-out, ongoing illicit substance use, diversion of DAM tablets and delinquency. Some patients that would otherwise qualify for HAT and mainly use illicit opioids intranasally may even be discouraged from entering HAT for these aforementioned reasons [4].

In order to address these clinical gaps, minimise harm, and improve treatment outcomes, a novel route of DAM administration (intranasal; IN) has been developed as an alternative to IV or oral DAM for patients in HAT. It has a similar onset of effect as IM administration and is thus likely to produce the desired “rush” [15–17]. Currently prescribed off-label in a small number of HAT patients, it seems to be associated with less adverse effects than IV, IM or SC injection and has been suggested as a harm reduction measure to reduce HCV infection rates [18, 19]. Intranasal HAT (n-HAT) was first described in a case-series and was suggested as an important risk-reduced rapid-onset alternative to other forms of HAT [20]. In a recently published case-report, IN DAM administration improved treatment adherence and health outcomes in a patient who craved the fast onset of effect of DAM but was unable to inject intravenously [19]. Similarly, patients in HAT comparing a one-off administration of IN DAM with IV DAM reported advantages for IN administration such as ease and convenience of use, avoidance of needle hazards, and reduced stigma [15]. Finally, overdose from nasal use occurs much less often than from IV use [21].

Few studies have investigated the pharmacokinetics and pharmacodynamics of IN DAM. However, these were conducted in pain patients [22, 23], “healthy” subjects with a history of heroin use but abstinent from opioids at the time of study [24, 25], and opioid-dependent subjects in oral OAT [17, 26] in doses much lower than those prescribed in HAT. Nevertheless, these studies demonstrated that after administration of IN DAM, considerable peak plasma concentrations of DAM and its metabolites are reached at a similar rate than IM administration [15, 17, 24, 25, 27]. One study that investigated the effects of 40 mg IN DAM versus 40 mg IV DAM in HAT patients suggests IN DAM to be effective in suppressing opioid withdrawal and acceptable to patients, if not preferable to injected use [15]. IN DAM has also been studied in paediatric pain patients, where it has been shown to be safe and effective [28].

To date, no study has investigated the clinical use of IN DAM in OAT. Despite the case report and case-series, n-HAT has never been systematically evaluated in a clinical study. To thoroughly evaluate the feasibility, safety, and acceptability of n-HAT, the first large-scale prospective multicentre observational study was developed.

## Methods

### Study design

The study is designed as an exploratory prospective multicentre clinical cohort study. Every HAT centre in Switzerland was invited to participate and 18 confirmed their participation. Given that there are 23 specialist outpatient centres offering HAT in Switzerland, this study is representative of the treatment system [29]. Within this treatment system, these outpatient centres provide HAT to around 1600 patients, which has been relatively constant within the last two decades [30]. Enrolled participants will switch from oral or injectable DAM (treatment as usual arm) to intranasal DAM. Follow-up will occur over three years, with assessments at baseline (t0), and after 4 (t4), 52 (t52), 104 (t104) and 156 (t156) weeks. These timepoints are standard follow-up periods when measuring retention in OAT [31]. Specifically, the first 4 weeks in OAT are particularly important as the risk of mortality is elevated when compared with the remainder of time receiving OAT [32]. Long-term retention in HAT has also been studied at similar time points (e.g., 2 years in Germany, 2 years in Spain, 4 years in the Netherlands) [33–35].

**Fig. 1:**
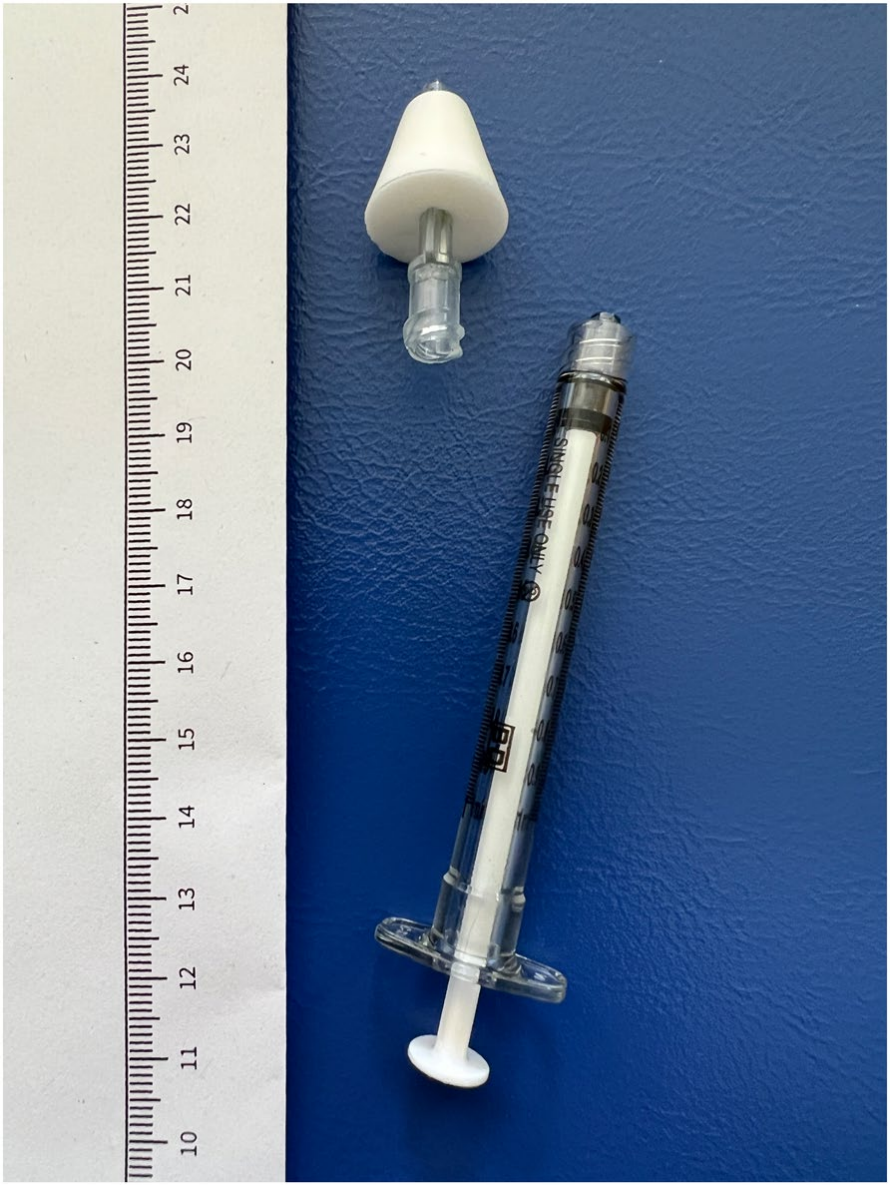
Barrel and mucosal atomisation device for IN DAM administration

Participants in n-HAT will receive Diaphin^®^ IV 100 mg/ml (DiaMo Narcotics, Thun), approved in Switzerland for IV treatment of OUD in the HAT setting. It will be administered into the nostrils with a mucosal atomisation device (Fig. 1). Atomisers are personalised, disinfected following each administration and replaced every 7 days. Just like conventional treatment with IV and IM DAM, long-acting oral opioids such as methadone or and slow-release oral morphine can be used as additional medication in order to prevent withdrawal. Participants will also be allowed to combine nasal with oral, IV and IM DAM. The dosing of IN DAM and of DAM through other routes of administration will remain an individual decision of patients and prescriber. Therefore, patients will not receive the same dosification, but rather receive an IN DAM dosification that align with their needs. There is no limitation on concomitant opioid and/or non-opioid medication to be prescribed. This study design is meant to be as comparable to the current Swiss HAT setting as possible.

This research project will be conducted in accordance with the study protocol, the Declaration of Helsinki [36], the principles of Good Clinical Practice (GCP), the Human Research Act (HRA) and the Human Research Ordinance (HRO) [37] as well as other locally relevant regulations. The study protocol received ethical approval from all responsible ethics committees. The study will be conducted in accordance with all applicable regulations and GCP-related guidelines.

### Study objectives and hypotheses

The study aims at investigating whether IN DAM is clinically feasible, acceptable to patients, and safe to use in HAT. The primary outcome measure (POM) is retention in HAT with the IN DAM route of administration after 4 weeks, 52 weeks, 104 weeks and 156 weeks. The secondary outcome measures (SOM) are prescriptions and routes of administration of other opioid agonists, illicit substance use, risk behaviours, delinquency, health and social functioning, treatment adherence, craving for opioids and other substances, satisfaction with prescribed opioid, subjective effects, quality of life, physical health, and mental health.

We hypothesise that patients receiving IN DAM in HAT will be retained in treatment with this route of administration over short and long time periods. Furthermore, patients are expected to report high satisfaction within IN DAM. Thirdly, we hypothesise IN DAM to be safe, the number of prescribed injection events to decrease and use of illicit opioids to decline.

### Study population

The inclusion criteria specific to this study comprise the ability to give informed consent, the patient’s wish to receive IN DAM during HAT participation and a medical indication for IN DAM. Swiss HAT centres additionally recommend a specialist examination of the nose and nasal cavities, but this is not mandatory for patients.

All other inclusion criteria are not specific to the study but correspond to the inclusion criteria of Swiss HAT and the guidelines for DAM treatment defined by the Swiss HAT institutions [29]. To be eligible for HAT in Switzerland, patients must be at least 18 years old, have a history of severe opioid dependence of more than 2 years, have a history of at least two unsuccessful conventional treatment attempts for opioid dependence (e.g., continued engagement in high-risk use behaviour) and have documented social or health problems as a result of opioid dependence [29]. We expect that most participants will be in HAT with other routes of administration prior to study inclusion.

Patient will not be eligible to participate in this study if they have severe cognitive impairment (e.g., dementia or substance-induced impairments) or insufficient language proficiency, rendering the reliable completion of the self-report forms/questionnaires impossible.

### Study procedures

Participants will be recruited only after the decision for nasal DAM is made by the participants and the responsible physician independently from study procedures. Patients will be informed that study participation is voluntary, that they may withdraw from the study at any time and that withdrawal of consent will not affect their subsequent medical assistance and treatment. Patients will be given at least a day to consider participation in the study. Study procedures comprises of assessments with questionnaires and structured interviews at baseline, 4, 52, 104, 156 weeks, and of a duration around one hour each. These are described below and in Table 1.

**Table 1:** Study assessments

*Study periods*	*Assessment 1 (Baseline)*	*Assessment 2*	*Assessments 3, 4 and 5*
*Time (weeks)*	*0*	*4*	*52, 104, 156*
*Inclusion/Exclusion Criteria*	*X*		
*Written Informed Consent*	*X*		
*Patient characteristics*	*X*		
*Medical History*	*X*		
*Retention*^*a*^	*X*	*X*	*X*
*Opioid agonist prescription*^*a*^	*X*	*X*	*X*
*Treatment adherence*^*a*^	*X*	*X*	*X*
*Frequency of visits*^*a*^	*X*	*X*	*X*
*Treatment Outcomes Profile (TOP)*^*b*^	*X*	*X*	*X*
*Health section of Opiate Treatment Index (OTI)*^*b*^*, including nose-related problems*	*X*	*X*	*X*
*Symptom-Checklist 27 (SCL-27)*^*b*^	*X*	*X*	*X*
*Adverse Events*^*b*^	*X*	*X*	*X*
*Opioid Agonist Scale*^*1,c*^	*X*	*X*	
*VAS Subjective effects*^*2,c*^	*X*	*X*	
*VAS Craving*^*c*^	*X*	*X*	*X*
*VAS Satisfaction*^*c*^	*X*	*X*	*X*

^1^20–30 min after DAM application

^2^10 min after DAM application

^a^Used to assess feasibility of IN DAM

^b^Used to assess safety of IN DAM

^c^Used to assess acceptability of IN DAM

Patients will remain in the study if they change route of administration of DAM and will continue assessments even if they no longer receive IN DAM until study completion after three years. Patients who switch from IN DAM to another DAM route of administration and subsequently switch back to IN DAM will be allowed to do so, as it is an observational study of clinical procedures. Short-term switches to other forms of DAM or OAT due to external circumstances such as vacations abroad (where DAM cannot be taken), illness, hospitalisations or similar will be considered as continuous IN treatment, as the basic prescription will not change. If a patient switches to another route of DAM administration as per prescription, this will be systematically assessed, and a change back to IN DAM as well.

### Primary outcome assessment

The primary outcome is retention in treatment with IN DAM after 4 weeks, 52 weeks, 104 weeks and 156 weeks. Success on primary outcome is defined as participants receiving IN DAM at each follow-up. Therefore, we will assess the prescription at the time point of assessment, and patients receiving IN DAM at the time point of assessment will be considered retained. However, since patient may switch to another route of DAM administration and then switch back to IN DAM within the follow-up periods, we will also separately calculate continuous IN DAM retention from “intermittent” IN DAM retention for patients changing and returning to and from other routes of DAM, and will assess time spent in prescription of IN DAM compared to other routes of administration.

### Secondary outcome assessment

Opioid agonist prescriptions including substance, galenics, dose and route of administration in the past 4 weeks will be assessed using patient’s the medical charts at each time point (t0, t4, t52, t104, t156).

Adherence to treatment and frequency of visits in the past 4 weeks will be assessed using the recorded scheduled and realised dispensings in the patient’s medical charts at each time point (t0, t4, t52, t104, t156).

Illicit substance use, delinquency, health and social functioning and quality of life will be assessed by the Treatment Outcome Profile (TOP) at each time point (t0, t4, t52, t104, t156). The TOP is a 20-item instrument which assesses substance use, injecting risk behaviour, and crime by self-report in the past 4 weeks [38]. Quality of life and social functioning will be assessed using the proposed 0-to-20-point scale of the TOP at each time point (t0, t4, t52, t104, t156).

Opioid craving in the past 7 days will be measured by visual analogue scales (VAS) at each time point (t0, t4, t52, t104, t156). Craving for alcohol, benzodiazepines and cocaine will be measured likewise. Acceptability of nasal DAM will be assessed by VAS on satisfaction with the different routes of administrations in DAM. Additionally, questions on the willingness to continue with the current prescription will be asked.

Subjective DAM effects will be measured by visual analogue scales (VAS) twice (t0 and t4), 10 min after DAM application. The VAS presents the participant a rating scale which represents the spectrum of “opioid high”, heroin-typical effects, good effects, bad effects, pleasure and feeling unwell: the left end indicates “none” while the right end indicates “extreme” effects.

Furthermore, subjective effects of opioids will be assessed using the self-reported German version of the Opioid Agonist Scale at baseline and 4 week follow-up point (t0 and t4) [39]. Between 20 and 30 min after DAM administration, participants will rate the extent to which they experienced each of 16 morphine-like symptoms on a five-point scale from “not at all” to “extremely”.

Physical health in the past 4 weeks will be assessed using the health section of the Opiate Treatment Index (OTI) at each time point (t0, t4, t52, t104, t156) [40]. Only the health section of the OTI will be used; the physical health section of the OTI is composed of items addressing signs and symptoms in major organ systems and injection-related health problems. An additional self-designed section regarding nose-related problems will also be included, inquiring for stuffed and/or runny nose, nasal burning or itching, nasal pain, nasal bleeding, alterations of smell or other problems.

Self-reported mental health symptoms in the last 7 days will be assessed using the Symptom-Checklist 27 (SCL-27) at each time point (t0, t4, t52, t104, t156). The SCL-27 is the short German version of the 90-item SCL-90 [41]. The SCL-27 is a commonly used reliable and valid instrument, consisting of six subscales and a general score.

Although this is an observational study and not a randomised controlled trial and the decision on which routes of administration will be used for DAM remains with clinicians and patients, we will assess safety with several different measures. First, study physicians will report any serious adverse events (SAEs) followed by immediate information of all study centres. Second, we will assess physical health and especially nasal health with the health section of the OTI and self-designed questions on nasal symptoms. Third, we will assess risk behaviour such as number of injection events inside and outside of treatment. AEs and SAEs are defined in the “Safety” section.

### Sample size and power calculation

We aim to include all patients willing to participate in the study. As this is an exploratory observational study, it is not possible at this point to exactly predetermine the final sample size, as it depends on the number of patients interested in and qualifying for n-HAT and consenting to participation in this clinical observation. We think that over the planned accrual time of two years, it is realistic to include between 100 and 200 participants who will try out IN DAM.

### Safety

Safety will be assessed by the appearance of adverse events (AEs) and serious adverse events (SAEs). AEs and SAEs will be monitored throughout the study. A serious event is defined as any adverse event where it cannot be excluded, that the event is attributable to the IN administration of DAM or the collection of health-related personal data, and which: (a) requires inpatient treatment or extends a current hospital stay, (b) results in permanent or significant incapacity or disability, or (c) is life-threatening or results in death.

If a serious adverse event related to study procedures occurs, the research project will be interrupted, and the Ethics Committee notified. If an event related to IN DAM administration is reported, all Swiss HAT centres will be informed. The decision whether ongoing IN DAM treatments are continued or not, however, will then be made independently of the study by the responsible physicians.

### Discontinuation

Participants will be withdrawn from the study if they withdraw informed consent or if they leave HAT. If a patient withdraws, only anonymised data gathered until this time point will be used. There will be no final examinations related to the study. Dropouts will be recorded as this relates to primary outcome (retention in treatment), but not followed up. Reasons for dropout will be assessed as this concerns acceptability of treatment. If a patient no longer receives IN DAM but remains in DAM treatment and does not withdraw informed consent, the scheduled visits will be conducted as planned.

### Data analysis

Statistical analysis of retention in treatment will be conducted with the appropriate statistical methods, depending on measurement-level of the variables (binary: t-test for repeated measures; interval: survival analysis).

The secondary outcomes will be assessed using Fisher’s exact, Wilcoxon–Mann Whitney, and interaction terms from Linear Mixed Models for binary, interval, and repeated measures, respectively.

## Discussion

This is the first study to investigate the clinical use of IN DAM in OAT. This prospective multicentre observational cohort study will do so by evaluating the feasibility, safety, and acceptability of n-HAT. Patients will be switched from conventional IV and oral DAM to IN DAM and followed-up over three years. Some preliminary findings at the first follow-up have showed positive results, with over 90% of participants still receiving IN DAM after four weeks [42].

This study was launched in order to meet the needs and minimise harm among two main groups of patients. Firstly, patients in treatment with injectable DAM (i-HAT) who suffer from injection-related problems such as deteriorated vein status, ulcerations, endocarditis, and abscesses. This is particularly prevalent in Switzerland and the EU as the opioid-dependent population is aging and has a long history of IV substance use. Secondly, patients who are eligible for HAT but do not respond to oral OAT, do not inject, and snort/sniff opioids, and are not eligible for i-HAT. This patient population is growing as preferred routes of administration for illicit opioids are shifting from injection to intranasal. This study will therefore evaluate IN DAM as a suitable option to address the needs of patients with severe OUD who do not respond to conventional OAT but who are unable/reluctant to inject or who primarily snort opioids. If IN DAM is found to be safe, feasible and acceptable, this study could pave the way for randomised controlled trials and in the longer-term approval of this new treatment option for HAT. It could reduce barriers to OAT, increase retention in treatment, and minimise harm in patients with a long history of injecting. Such findings supporting the use n-HAT as another treatment strategy would help move the treatment of OUD away from an ineffective “one size fits all” approach [1].

Internationally, the intranasal route of administration may provide new paths for treatment and research. For instance, in countries where HAT is available (e.g., the Netherlands, Germany, England, Luxemburg and Denmark), findings from this study, if supportive of n-HAT, may encourage similar research efforts and the development of implementation strategies. For countries where HAT is not available or only to a very limited extent (e.g., Canada, the United States), IN DAM as a safe, feasible, and acceptable new treatment option for OUD may help advocate for the implementation of HAT, increase the accessibility of HAT, and combat the significant stigma associated with substance use treatment. As it relates to fentanyl and fentanyl analogues in North America, a similar approach with nasal fentanyl could be promising for people who deliberately inject or sniff fentanyl, but much more research is needed on this front (43).

### Strengths and limitations

This study has several limitations. Firstly, it is not a randomised controlled trial and has the inherent weaknesses of an observational study. Secondly, most study participants will already be patients in HAT before being started on IN DAM and thus familiar with the clinic procedures and DAM prescription. This does however make them well suited to describe the subjective effects of the nasal route of administration DAM and compare them to their previous routes of administration. Thirdly, the patients recruited to participate in this study were interested in IN DAM, as the decision to start IN DAM was made independently from study procedures. This therefore limits the generalisability of the findings, particularly as it relates to the acceptability of IN DAM among other patients. Finally, some of our outcome assessments are based on self-report and are subject to recall bias and social desirability (e.g., delinquency and criminality). Despite the limitations, the present study has major strengths. It is a national multicentre study with a moderate sample size that will be easily translatable to clinical practice.

## Conclusion

This is the first large-scale prospective observational cohort study on the intranasal administration of DAM in a clinical setting among individuals receiving HAT. The findings derived from this study will produce the first major body of clinical evidence on the feasibility, safety, and acceptability of n-HAT. If the findings from this study align with the hypotheses, n-HAT may become a viable option for patients with injection-related complications, or for non-injecting opioid-dependent patients failing to respond to oral OAT. This would be a big step forward in increasing the accessibility of OAT for individuals with OUD and in promoting the use of and advocating for HAT in countries where this treatment form is currently unavailable. More research efforts are needed to more systematically assess n-HAT, and how n-HAT can be used together with other opioid agonist medications for optimal treatment outcome.

## Data Availability

Not applicable: no datasets were generated or analysed during the current study.

## Abbreviations

AE: Adverse events
DAM: Diacetylmorphine
HAT: Heroin-assisted treatment
i-HAT: Injectable heroin-assisted treatment
IM: Intramuscular
IN: Intranasal
IV: Intravenous
n-HAT: Nasal heroin-assisted treatment
OAT: Opioid agonist treatment
OTI: Opiate treatment index
OUD: Opioid use disorder
POM: Primary outcome measure
PWUD: People who use drugs
SAE: Serious adverse events
SC: Subcutaneous
SCL-27: Symptom-checklist 27
SOM: Secondary outcome measure
TOP: Treatment outcomes profile
VAS: Visual analogue scale

## References

[1] Nordt C, Vogel M, Dey M, Moldovanyi A, Beck T, Berthel T (2019). One size does not fit all—evolution of opioid agonist treatments in a naturalistic setting over 23 years. Addiction.

[2] Strang J, Groshkova T, Uchtenhagen A, van den Brink W, Haasen C, Schechter MT (2015). Heroin on trial: systematic review and meta-analysis of randomised trials of diamorphine-prescribing as treatment for refractory heroin addiction. Br J Psychiatry.

[3] Strasser J, Vogel M (2013). Die Zukunft der heroingestützten Behandlung aus klinischer Sicht in der Schweiz [The future of heroin assisted treatment from a clinical point of view]. Suchtmedizin Forschung Praxis.

[4] Vogel M, Strasser J (2017). Alternativen in der Behandlung mit im Zentralnervensystem rasch anflutenden opioiden. Suchtmedizin.

[5] Dürsteler-MacFarland KM, Vogel M, Wiesbeck GA, Petitjean SA (2011). There is no age limit for methadone: a retrospective cohort study. Subst Abuse Treat Prev Policy.

[6] Vogel M, Dürsteler K, Strasser J, Schmid O, Müller E, Himmelheber P (2015). Injektionen in die Leistenvene: Prävalenz und Umgang in heroingestützter Behandlung. Suchtmedizin.

[7] Larney S, Peacock A, Mathers BM, Hickman M, Degenhardt L (2017). A systematic review of injecting-related injury and disease among people who inject drugs. Drug Alcohol Depend.

[8] Hope VDD, Parry JVV, Ncube F, Hickman M (2016). Not in the vein: ‘missed hits’, subcutaneous and intramuscular injections and associated harms among people who inject psychoactive drugs in Bristol, United Kingdom. Int J Drug Policy.

[9] Meyer M, Eichenberger R, Strasser J, Dürsteler KM, Vogel M (2021). «One prick and then it´s done»: a mixed-methods exploratory study on intramuscular injection in heroin-assisted treatment. Harm Reduct J..

[10] Girardin F, Rentsch KM, Schwab MA, Maggiorini M, Pauli-Magnus C, Kullak-Ublick GA (2003). Pharmacokinetics of high doses of intramuscular and oral heroin in narcotic addicts. Clin Pharmacol Ther.

[11] Gyr E, Brenneisen R, Bourquin D, Lehmann T, Vonlanthen D, Hug I (2000). Pharmacodynamics and pharmacokinetics of intravenously, orally and rectally administered diacetylmorphine in opioid dependents, a two-patient pilot study within a heroin-assisted treatment program. Int J Clin Pharmacol Ther.

[12] Klous MG, Van den Brink W, Van RJM, Beijnen JH (2005). Development of pharmaceutical heroin preparations for medical co-prescription to opioid dependent patients. Drug Alcohol Depend.

[13] Gesundheitsdepartement Basel-Stadt. Bericht Kontakt- und Anlaufstellen Basel-Stadt 2016—Statistische Auswertung der Besucherzahlen, Konsumhäufigkeiten und konsumierten Substanzen. Basel. 2017.

[14] EMCDDA. European Drug Report (2017). Luxembourg.

[15] Mitchell TB, Lintzeris N, Bond A, Strang J (2006). Feasibility and acceptability of an intranasal diamorphine spray as an alternative to injectable diamorphine for maintenance treatment. Eur Addict Res.

[16] Rook EJ, Van Ree JM, Van Den Brink W, Hillebrand MJX, Huitema ADR, Hendriks VM (2006). Pharmacokinetics and pharmacodynamics of high doses of pharmaceutically prepared heroin, by intravenous or by inhalation route in opioid-dependent patients. Basic Clin Pharmacol Toxicol.

[17] Comer SD, Collins ED, MacArthur RB, Fischman MW (1999). Comparison of intravenous and intranasal heroin self-administration by morphine-maintained humans. Psychopharmacology.

[18] Des Jarlais DC, Hagan H, Arasteh K, McKnight C, Semaan S, Perlman DC (2011). Can intranasal drug use reduce HCV infection among injecting drug users?. Drug Alcohol Depend.

[19] Meyer M, Westenberg JN, Strasser J, Dürsteler KM, Lang UE, Krausz M (2022). Nasal administration of diacetylmorphine improved the adherence in a patient receiving heroin-assisted treatment. Harm Reduct J.

[20] Vogel M, Köck P, Strasser J, Kalbermatten C, Binder H, Dürsteler KM (2022). Nasal opioid agonist treatment in patients with severe opioid dependence: a case series. Eur Addict Res.

[21] Darke S, Ross J (2000). Fatal heroin overdoses resulting from non-injecting routes of administration, NSW, Australia, 1992–1996. Addiction.

[22] Hallett A, O’Higgins F, Francis V, Cook TM (2000). Patient-controlled intranasal diamorphine for postoperative pain. Anaesthesia.

[23] Ward M, Minto G, Alexander-Williams JM (2002). A comparison of patient-controlled analgesia administered by the intravenous or intranasal route during the early postoperative period. Anaesthesia.

[24] Cone EJ, Holicky BA, Grant TM, Darwin WD, Goldberger BA (1993). Pharmacokinetics and pharmacodynamics of intranasal “snorted” heroin. J Anal Toxicol.

[25] Skopp G, Ganssmann B, Cone EJ, Aderjan R, Skoppl G, Ganssmann B (1997). Plasma concentrations of heroin and morphine-related metabolites after intranasal and intramuscular administration. J Anal Toxicol.

[26] Comer SD, Collins ED, Fischman MW (1997). Choice between money and intranasal heroin in morphine-maintained humans. Behav Pharmacol.

[27] Kendall JM, Latter VS (2003). Intranasal diamorphine as an alternative to intramuscular morphine: pharmacokinetic and pharmacodynamic aspects. Clin Pharmacokinet.

[28] Kendall J, Maconochie I, Wong ICK, Howard R, DIASAFE study on behalf of the D (2015). A novel multipatient intranasal diamorphine spray for use in acute pain in children: pharmacovigilance data from an observational study. Emerg Med J..

[29] Federal Office of Public Health FOPH. Diacetylmorphine-assisted (heroin-assisted) treatment [Internet]. 2022 [cited 2023 Mar 9]. https://www.bag.admin.ch/bag/en/home/gesund-leben/sucht-und-gesundheit/suchtberatung-therapie/substitutionsgestuetzte-behandlung.html.

[30] Gmel G, Labhart F, Maffli E. Heroingestützte/diacetylmorphingestützte Behandlung in der Schweiz—Resultate der Erhebung 2020 (Forschungsbericht Nr. 135). 2021.

[31] Klimas J, Hamilton MA, Gorfinkel L, Adam A, Cullen W, Wood E (2021). Retention in opioid agonist treatment: a rapid review and meta-analysis comparing observational studies and randomized controlled trials. Syst Rev..

[32] Santo T, Clark B, Hickman M, Grebely J, Campbell G, Sordo L (2021). Association of opioid agonist treatment with all-cause mortality and specific causes of death among people with opioid dependence: a systematic review and meta-analysis. JAMA Psychiatry.

[33] Verthein U, Bonorden-Kleij K, Degkwitz P, Dilg C, Köhler WK, Passie T (2008). Long-term effects of heroin-assisted treatment in Germany. Addiction.

[34] Blanken P, Hendriks VM, van Ree JM, van den Brink W (2010). Outcome of long-term heroin-assisted treatment offered to chronic, treatment-resistant heroin addicts in the Netherlands. Addiction.

[35] Oviedo-Joekes E, March JC, Romero M, Perea-Milla E (2010). The Andalusian trial on heroin-assisted treatment: A 2 year follow-up. Drug Alcohol Rev.

[36] World Medical Association (2013). WMA declaration of Helsinki—ethical principles for medical research involving human subjects—WMA—the world medical association. JAMA..

[37] The Swiss Federal Council (2018). Ordinance on human research with the exception of clinical trials (HRO). Swiss Med Week.

[38] Marsden J, Farrell M, Bradbury C, Dale-Perera A, Eastwood B, Roxburgh M (2008). Development of the treatment outcomes profile. Addiction.

[39] Preston KL, Bigelow GE, Bickel W, Liebson IA (1987). Three-choice drug discrimination in opioid-dependent humans: hydromorphone, naloxone and saline. J Pharmacol Exp Ther.

[40] Darke S, Ward J, Hall W, Heather N, Wodak A (1991). The opiate treatment index manual technical report 11.

[41] Hardt J, Egle UT, Kappis B, Hessel A, Brähler E (2004). Die Symptom-Checkliste SCL-27. PPmP Psychotherapie Psychosomatik Medizinische Psychol..

[42] Vogel M, Meyer M, Westenberg JN, Kormann A, Simon O, Salim R (2023). Safety and feasibility of intranasal heroin-assisted treatment: 4-week preliminary findings from a Swiss multicentre observational study. Harm Reduct J.

[43] Krausz RM, Westenberg JN, Vogel M (2022). Addressing fentanyl use disorder with fentanyl-assisted treatment. Lancet Psychiatry.

